# Impact of deep water running in interval training (DWR-IT) on body composition, functional capacity, and quality of life in overweight adults: study protocol for a randomized controlled trial

**DOI:** 10.1186/s13063-019-3618-7

**Published:** 2019-09-11

**Authors:** Bruna Pianna, Bianca Christianini Moreno, Caroline Aquino de Souza, Thais Fernanda Bôscoa, Guilherme Eleutério Alcalde, Silvia Regina Barrile, Camila Gimenes, Bruno Martinelli, Antonio Roberto Zamunér, Bruna Varanda Pessoa-Santos, Eduardo Aguilar Arca

**Affiliations:** 1 Pró-Reitoria de Pesquisa e Pós-Graduação da Universidade do Sagrado Coração, Street Irmã Arminda, 10-50, Jardim Brasil, Bauru, SP Brazil; 2 Course Physical therapy of the Universidade do Sagrado Coração, Bauru, SP Brazil; 3 Departamento de Kinesiología, Universidad Católica del Maule, Talca, Maule VII Región Chile; 4 Professor of the Universidade de Cuiabá, Cuiabá-MT, Brazil

**Keywords:** Hydrotherapy, Obesity, Body composition, Middle-aged, Physical fitness

## Abstract

**Background:**

Interval training in deep water running (DWR-IT) is a training method to improve cardiovascular fitness, functional health, and quality of life and to help control body weight. Its main advantages are the reduction of joint overload and a low risk of musculoskeletal injuries. The aim of the study is to investigate the influence of DWR-IT on functional capacity, body composition, and quality of life of overweight middle-aged adults.

**Methods:**

This is a randomized controlled, two-arm, open, parallel clinical trial with overweight adults. Volunteers will be allocated to a water group (WG), which will be submitted to the intervention, or a control group, which will not be subjected to any kind of intervention. The evaluation will be composed of anamnesis, electrical bioimpedance, six-minute walk test (6MWT), questionnaire on the Impact of Weight on Quality of Life-lite (IWQOL-LITE), Pittsburgh Sleep Quality Index, Epworth Sleepiness Scale, chair stand test, arm curl test, and food frequency questionnaire. The DWR-IT will last for 12 weeks, systematically increasing the intensity and training volume.

**Discussion:**

The objective of this clinical trial is to evaluate the effect of DWR-IT on overweight adults. The study is guided through practice based on scientific evidence for the use of training and aquatic rehabilitation. It is expected that after 12 weeks of aquatic intervention there will be a decrease in body fat by about 10%, evaluated by electrical bioimpedance, an increase of about 25% of cardiorespiratory endurance, evaluated by 6MWT, and an improvement of about 25% of physical function domains, self-esteem, distress in public places, and work, analyzed by IWQOL-LITE in the WG.

**Trial registration:**

The study protocol was published in the Brazilian Registry of Clinical Trials (ReBEC) on June 16, 2016. Registration number: RBR-6dmh7d.

**Electronic supplementary material:**

The online version of this article (10.1186/s13063-019-3618-7) contains supplementary material, which is available to authorized users.

## Background

Obesity is a serious public health problem in Brazil and worldwide [1]. It has been defined as a chronic disease characterized by the excessive accumulation of body fat in the adipose tissue [2, 3]. Obesity affects 13% of the world’s adult population and if this trend continues, the percentage can reach 20% by 2025 [4]. In Brazil, statistics are also alarming since in 10 years the prevalence rates of overweight have increased [5]. Overweight and obesity are associated with increased morbidity and mortality. Some studies showed that overweight was associated with twice the risk of developing cardiometabolic disease—such as coronary artery disease, cerebral apoplexy, and high blood pressure, and type 2 diabetes mellitus—and other diseases such as endometrial and colorectal cancer, sleep apnea, low self-esteem and eating disorders are also on the rise because of obesity [6].

Obesity and overweight are ewlated to musculoskeletal pain and injury, biomechanical adaptions as a result of the sheer bulk and force of increased fat mass affected locomotion, balance, and strength. Obesity also negatively affects cardiorespiratory function, a fact that promotes the decline of quality of life and sleep and activities of daily living [6–8].

The regular practice of physical activity has been shown to be an efficient strategy to increase energy expenditure and consequently to minimize the effect of excess body weight on quality of life and sleep [6]. Thus, it has been considered a primary component for promoting the physical, functional, psychological, and cognitive health of overweight and obese individuals [7–10]. The American College of Sports Medicine recommend as a component of weight management for prevention of weight gain, for weight loss, a minimum of 150 min-week to 250, 250 min-week of moderate-intensity physical activity [11]. However, it is difficult for an obese person to participate because of increased excessive joint overload, pain, and risk of musculoskeletal injuries [12–14].

Aquatic exercise is highly recommended because of its numerous benefits to the human body through the use of the water’s buoyancy, which can help decrease body fat while maintaining superior stability compared with ground exercises [15]. The deep water running in interval training (DWR-IT) is a new approach in the field of aquatic exercise to control obesity and aims to increase energy expenditure, improve cardiovascular fitness, and reduce body fat percentage [16].

DWR-IT is an aquatic aerobic exercise performed in relatively deep pools and consists of simulated running while wearing a floatation vest, which serves to keep the body in an upright position and helps to prevent contact between the feet and the bottom of the pool [17]. The main advantages of the DWR-IT technique are buoyancy and lack of weight-bearing, minimizing the repeated stress and impact experienced in running on land, and low risk of musculoskeletal injuries. In addition, the individual can perform the same amount of work intermittently and with the same intensity as continuous exercise; however, the degree of fatigue after interval training is considerably smaller and more pleasant for this population [18].

However, little is known about the chronic effects of DWR-IT performed in a heated pool (32 °C), in the body composition evaluated by electrical bioimpedance and functional capacity measured by the six-minute walk test (6MWT) in overweight and middle-aged obese.

Given the above, the proposal of the present study for the advancement of scientific knowledge in the area of aquatic training and rehabilitation in overweight and obese women becomes relevant. Accordingly, the objective of the study will be to investigate the effect of the DWR-IT program on the body composition, functional capacity, and quality of life of overweight adults.

## Methods

**Fig. 1:**
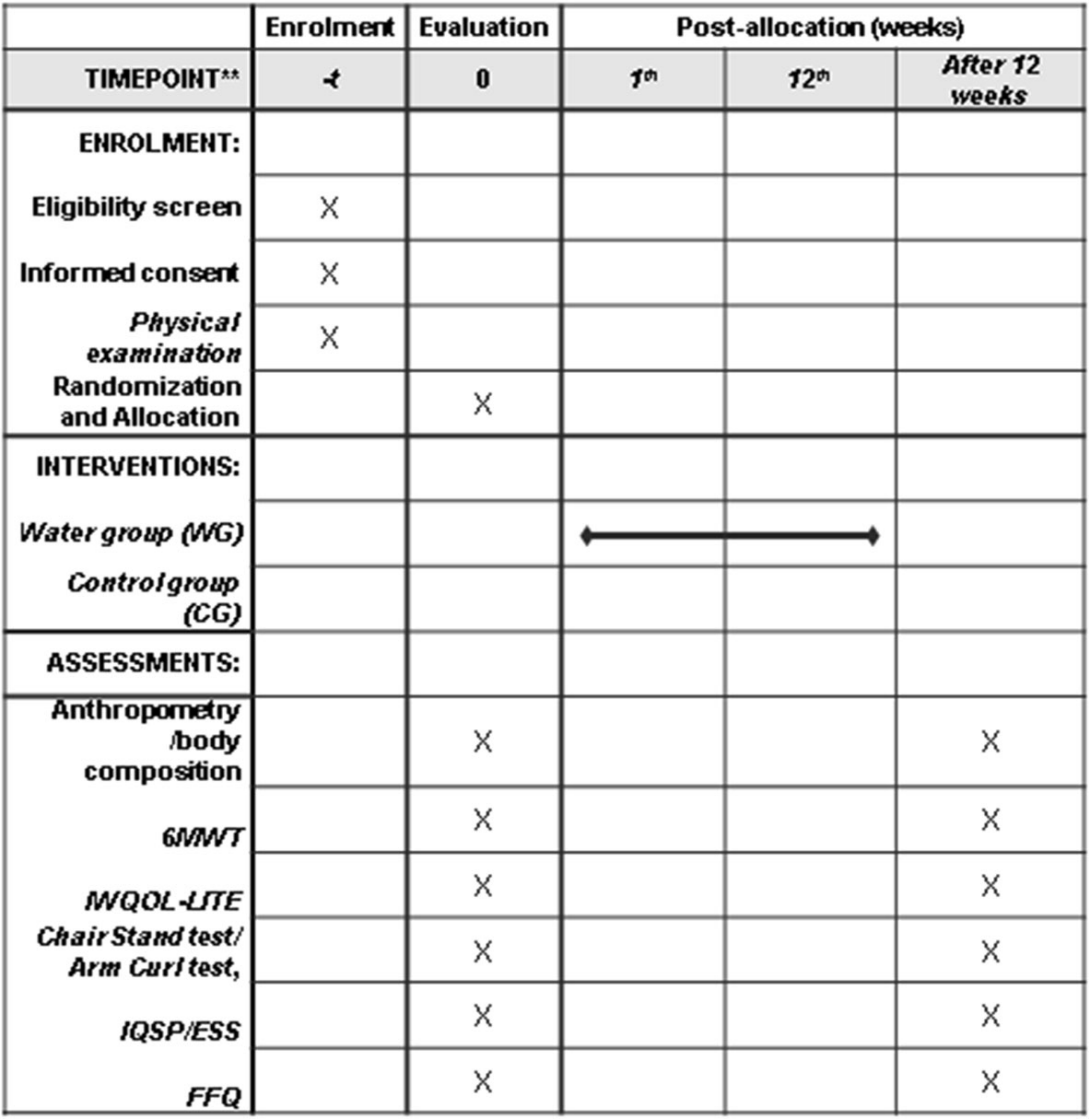
Standard Protocol Items: Recommendations for Interventional Trials (SPIRIT). Information about the selection, recruitment and evaluations carried out in each period. *Abbreviations*: *6MWT* six-minute walk test, *BMI* body mass index, *CG* control group, *ESS* Epworth Sleepiness Scale, *FFQ* food frequency questionnaire, *PSQI* Pittsburgh Sleep Quality Index, *IWQOL-LITE* questionnaire on the Impact of Weight on Quality of Life-lite, *WG* water group

The protocol was developed in accordance with the Standard Protocol Items: Recommendations for Interventional Trials (SPIRIT) and Consolidated Standards of Reporting Trials (CONSORT) guidelines and checklists. See Additional file 1 for a SPIRIT checklist and Fig. 1 for the recommended SPIRIT figure.

### Overview of research design

This protocol is a two-arm parallel randomized controlled therapeutic clinical trial recruiting overweight and obese volunteers.

### Registry of clinical trials

The study protocol was registered and published in the Brazilian Registry of Clinical Trials (ReBEC) (registration number: RBR-6dmh7d).

### Availability of data and material

Evaluations will be carried out at the Research Laboratory for Physical Therapy, and the intervention program will be held at the Therapeutic Pools Laboratory from the Universidade do Sagrado Coração, Bauru, São Paulo, Brazil. The researcher himself will be responsible for the assessment, reassessment, and supervision of the intervention program.

### Inclusion criteria

Volunteers of both sexes, between 39 and 59 years old (middle-aged), classified as pre-obesity (body mass index (BMI) ≥25 kg/m^2^ to 29.9 kg/m^2^) and obesity (BMI ≥30 kg/m^2^ to 34.9 kg/m^2^) will participate in the study [19]. All volunteers must present a medical prescription attesting the absence of cardiovascular disease that is restrictive to practice the aquatic physical training.

### Exclusion criteria

The exclusion criteria included any history of neuromuscular and cardiorespiratory disease (self-declared) and any contraindications of aquatic physiotherapy: hydrophobia, cutaneous wounds, and infectious diseases [20]. Volunteers may not have participated in another physical training program for at least two months from the beginning of the data collection and may not participate in another training or nutritional monitoring program during the aquatic intervention. For those who use anti-hypertensive drugs and hormone replacement therapy, a change in medication class or dose will not be allowed. As a criterion of adherence after the training period, data from samples that did not obtain at least 80% frequency in the sessions will be excluded.

### Baseline characteristics

After recruitment and initial screening, details covering demographic data, lifestyle, medications, identification of other diseases, anthropometric assessments, and cardiovascular variables—i.e., blood pressure and heart rate (HR)—will be collected.

### Randomization and allocation

**Fig. 2:**
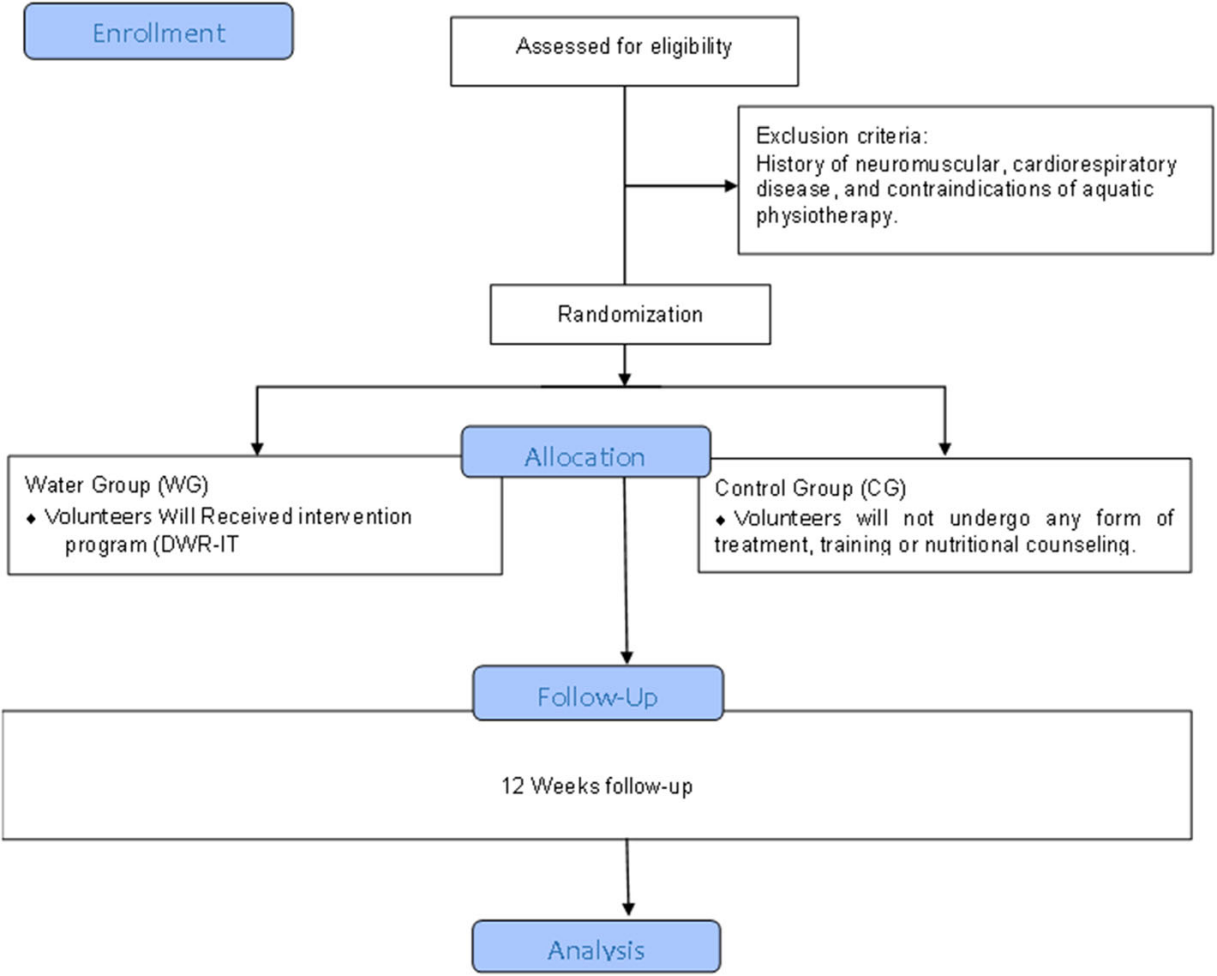
Flow diagram of the randomized clinical trial. Diagram of the randomized clinical trial. Detailed information about volunteer recruitment and selection and follow-up during the 12 weeks. *Abbreviations*: *6MWT* six-minute walk test, *BMI* body mass index, *CG* control group, *ESS* Epworth Sleepiness Scale, *FFQ* food frequency questionnaire, *PSQI* Pittsburgh Sleep Quality Index, *IWQOL-LITE* questionnaire on the Impact of Weight on Quality of Life-lite, *WG* water group

Randomization will be performed after baseline assessments. Sequentially numbered opaque sealed envelopes will be prepared ahead of time and randomly assigned by a computer-generated table of random numbers in a 1:1 proportion. A person blinded to the study protocol will perform the randomization and provide the group assignment to the treating physical therapist. Volunteers will be allocated to the water group, which will be submitted to the 12-week DWR-IT program, or to the control group, which will not be submitted to intervention and will be instructed not to engage in any kind of structured physical exercise training and to keep the life activities identified at baseline. The International Physical Activity Questionnaire (IPAQ) will be applied before and after the intervention in order to assess the level of physical activity. Failure to follow the recommendations is an exclusion criterion in the study (Fig. 2).

### Procedures

#### Anthropometry measures and body composition

Body mass (in kilograms) will be measured by using a digital anthropometric scale (Filizola^®^, Coats Corrente, São Paulo, SP, Brazil). A stadiometer (0.5 cm accuracy) will be used to measure the participant’s height (in meters). BMI will be calculated by using body weight measurements and height by the equation: BMI = kg/m^2^ [20].

An inextensible cellulose anthropometric tape will be used to measure the abdominal circumference [21]. Body composition will be evaluated by using Biodynamics TBW^®^ equipment (model 310, version 8.01; Biodynamics Corp., Shoreline, WA, USA) [22].

#### Six-minute walk test

This test evaluates the maximum distance travelled in six min while walking on the demarcated distance measures. There will be two tests with a 30-min interval between them in order to rule out learning and anxiety variables. The test with the greater distance achieved will be picked for analysis [23, 24].

#### Short form of impact of weight on quality of life

This consists of 31 items divided into five domains: physical function, self-esteem, sexual life, distress in public places, and work. The total score of each domain varies from 0 to 100; “0” corresponds to the worst general health condition and “100” corresponds to the best overall health status [25].

#### Muscle strength and endurance test

The chair stand test (CST) evaluates inner limb muscle strength and endurance and consists of 30-s sit-to-stand repetition movements in a chair without using the upper limbs [24]. The arm curl test (ACT) evaluates upper limb strength and endurance and consists of performing the greatest number of elbow flexion and extension for 30 s with 2-kg dumbbells for women and 4-kg dumbbells for men [26]. After both tests are performed, the number of repetitions will be recorded.

#### Sleep quality

The Pittsburgh Sleep Quality Index (PSQI), composed of 19 items, will be applied. The maximum score of this instrument is 21 points; scores above 5 are indicative of poor sleep quality [26]. The Epworth Sleepiness Scale (ESS) evaluates the likelihood of falling asleep while performing daily activities. Results between 0 and 10 points indicate absence of drowsiness; between 10 and 16 points, mild drowsiness; between 16 and 20 points, moderate drowsiness; and between 20 and 24 points, severe somnolence [27].

#### Food frequency questionnaire

This instrument is composed of a list of foods, distributed into 14 food groups, taking into account the frequency and quantity of each food consumed [28].

#### Intervention and follow-up periods

The intervention program will comprise 36 sessions, three times a week (in alternate days) during the morning, for 12 weeks, performed in a heated pool (32 °C). Sessions will be held in groups of no more than four volunteers and will be supervised by at least two therapists.

#### Water group (intervention group)

For the DWR-IT program, volunteers will use a Deep Runner vest (Floty^®^, Indaiatuba, SP, Brazil) and cardiac frequency meter (Polar^®^, Electro, Oi, Finland) for HR control and monitoring. To calculate the intensity of DWR-IT, the heart rate maxima (HRmax) will be recorded in the terrestrial environment by using the following equation: HRmax = 220 – age [29]. Next, the volunteers will remain immersed in the orthostatic position with water at the level of the sternum. After 5 min of rest, the HR will be measured. Afterwards, the immersion bradycardia (ΔHR), which in turn depends on the depth, temperature, and posture during the exercise, will be calculated [29]. The following formula HRmax - ΔHR will be applied to calculate the heart rate of volunteers in the aquatic environment, the following HRmax - ΔHR equation will be used [30]. During each DWR-IT session, the subjective perception of effort will be monitored and recorded [31].

**Fig. 3:**
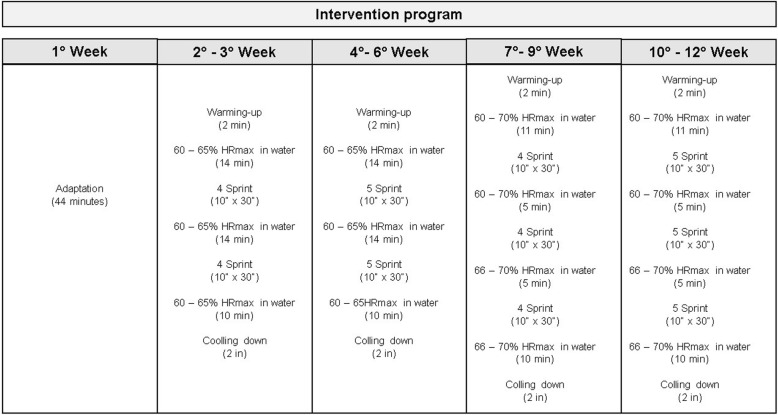
Description of aquatic therapy program. Detailed information about the intervention protocol, with the sprints and intensities determined for each intervention week

- First week: adaptation to a liquid environment and DWR-IT learning (44 min).
- Second and third week: warm-up of 2 min, then the exercise with an intensity of 60% to 65% of HRmax in the water, lasting 34 min. Two moments of sprints with 10 s for 30 s of rest (four series) were performed between the continuous exercises. After the training, 2 min of cool down was performed.
- Fourth to sixth week: the same procedures were performed as described in item b, but the continuous exercises lasted 33 min and five sprints were performed.
- Seventh to ninth week: the same procedures described in item b were performed, but the continuous exercises had an intensity of 66% to 70% of HRmax in the water, lasting 31 min. Three moments of sprints (four series) were performed among the continuous exercises.
- Tenth to twelfth week: the same procedures were performed as described in item b, but the continuous exercises had an intensity of 66% to 70% of the HRmax in the water, lasting 30 min, and three sprints (five series). Figure 3 shows the schematic diagram of the DWR-IT program.

#### Control group

The control group will not undergo any form of treatment. Volunteers will be instructed not to change their daily usual activities or diet or participate in other physical activities. During intervention, the volunteers will be monitored by phone calls from the researchers every week, and IPAQ and food frequency questionnaire (FFQ) will be used to monitor and control the level of physical activity and the frequency and quantity of food consumed; both will be applied before intervention, at week 6, and after the intervention period. If there are changes in dietary habits or level of physical activity, the volunteers will be excluded from the study.

#### Sample size

The sample size was performed on the basis of a pilot study, conducted previously with middle-aged women submitted to a DRW protocol [32]. Sample size calculation was performed by using G*Power version 3.1 software (Heinrich-Heine-Universität Düsseldorf, Düsseldorf, Germany). The maximum distance covered in 6MWT assessed before (566.2 ± 51.8 m) and after (625.1 ± 73.6 m) the DWR program was considered the main outcome. Significance level was set at 5% with 80% power. A sample of 12 participants per group was suggested. Given a possible 20% dropout rate, 15 participants will be required per group, thus totaling 30 participants.

#### Analysis

The normality and homogeneity of data variance will be verified through the Shapiro–Wilk and Levene tests, respectively. For the variables with normal distribution, the two-way mixed-design analysis of variance (ANOVA) test (a repetition factor and a group factor), followed by Bonferroni post hoc, will be applied. For variables with non-normal distribution, the Wilcoxon tests will be used to compare the differences between times and the Mann–Whitney test to compare differences between groups. In this case, the Bonferroni adjustment will be used *a priori*. In all tests, the significance level of 5% will be considered. The effect size will be calculated by using Cohen’s d, and the results interpreted on the basis of Cohen as follows: small (0.21–0.49), medium (0.50–0.79), or large (≥0.80) [33].

## Discussion

The objective of the DWR-IT program will be to investigate the influence of the 12-week DWR-IT program on body composition, functional capacity, and quality of life of overweight adults. DWR-IT can be very beneficial to the health of overweight people since it increases energy expenditure besides promoting positive effects on aerobic capacity and cardiovascular function and improvement of perception of quality of life and sleep in overweight and obese subjects submitted to training [32, 34]. It is believed that in addition to having the aforementioned outcomes, the DWR-IT program may be beneficial to the health of overweight middle-aged adults since the physical properties and the heated temperature of the water promote pain relief, reduction of joint overload, and low risk of musculoskeletal injuries [35–37].

Pasetti; Gonçalves; Padovani [37] subjected 30 obese middle-aged women to 12 weeks of DWR (47 min per session, three times per week, and water temperature between 28 °C and 29 °C) in two different modalities: continuous training (*n* = 12, 45.3 ± 6.3 years) with an intensity of 70–85% of resting HR and interval training (TI; *n* = 18; 46.6 ± 7.9 years) with an intensity between 70% and 75% of HR of rest. Body composition was assessed by using adipometry, and the World Health Organization Quality of Life BREF (WHOQOL-BREF) was used for quality-of-life analysis. After the aquatic training period, there was a reduction in body density and percentage of fat and improvement in quality of life in both types of training [37].

However, in spite of the benefits promoted by DWR, no randomized clinical trials on DWR-IT were found in the literature in the past five years that investigated functional capacity, muscle strength, sleep quality, or quality of life analyzed by a specific instrument for this population. In addition, in the aforementioned clinical trials, adequate methodologies for the control of the intensity of the aquatic exercises were not used since the physiological adaptations were not considered during immersion in the cardiovascular system, particularly in the HR. Finally, food consumption (pre- and post-intervention) was not recorded, a fact that may interfere in the responses of the variables related to body composition [37–40].

This study presents some strengths that in previous studies were not considered, such as the presence of the control group and use of specific instruments for the obese population. For the prescription of exercise intensity in the aquatic environment should take into account the help of HR for the prescription of intensity in the aquatic environment, thus avoiding cardiovascular overloaded of the control group, use of specific instruments for the obese population.

Therefore, the present study will provide clinical evidence that the DWR-IT program is effective in improving functional capacity, muscle strength, sleep quality, and quality of life in the obese. Thus, DWR is an alternative aquatic exercise to be considered by professionals in order to develop effective interventions for adults with obesity.

### Trial status

In data collection.

## Additional file

Additional file 1: SPIRIT (Standard Protocol Items: Recommendations for Interventional Trials) 2013 Checklist: Recommended items to address in a clinical trial protocol and related documents*. (DOC 121 kb)

## Abbreviations

ΔHR: Immersion bradycardia
BMI: Body mass index
DWR-IT: Deep water running in interval training
HR: Heart rate
HRmax: Heart rate maxima
IPAQ: International Physical Activity Questionnaire
SPIRIT: Standard Protocol Items: Recommendations for Interventional Trials
